# Supplementary material for: Cross-site reproducibility of human cortical organoids reveals consistent cell type composition and architecture
Source: bioRxiv. 2023 Jul 29:2023.07.28.550873. Preprint. [Version 1] doi: 10.1101/2023.07.28.550873 (PMC10402155; doi:10.1101/2023.07.28.550873)
Supplement: Supplement 5 [file NIHPP2023.07.28.550873v1-supplement-5.pdf]

bioRxiv preprint doi: https://doi.org/10.1101/2023.07.28.550873; this version posted July 29, 2023. The copyright holder for this preprint (which was not certified by peer review) is the author/funder, who has granted bioRxiv a license to display the preprint in perpetuity. It is made available under aCC-BY 4.0 International license.

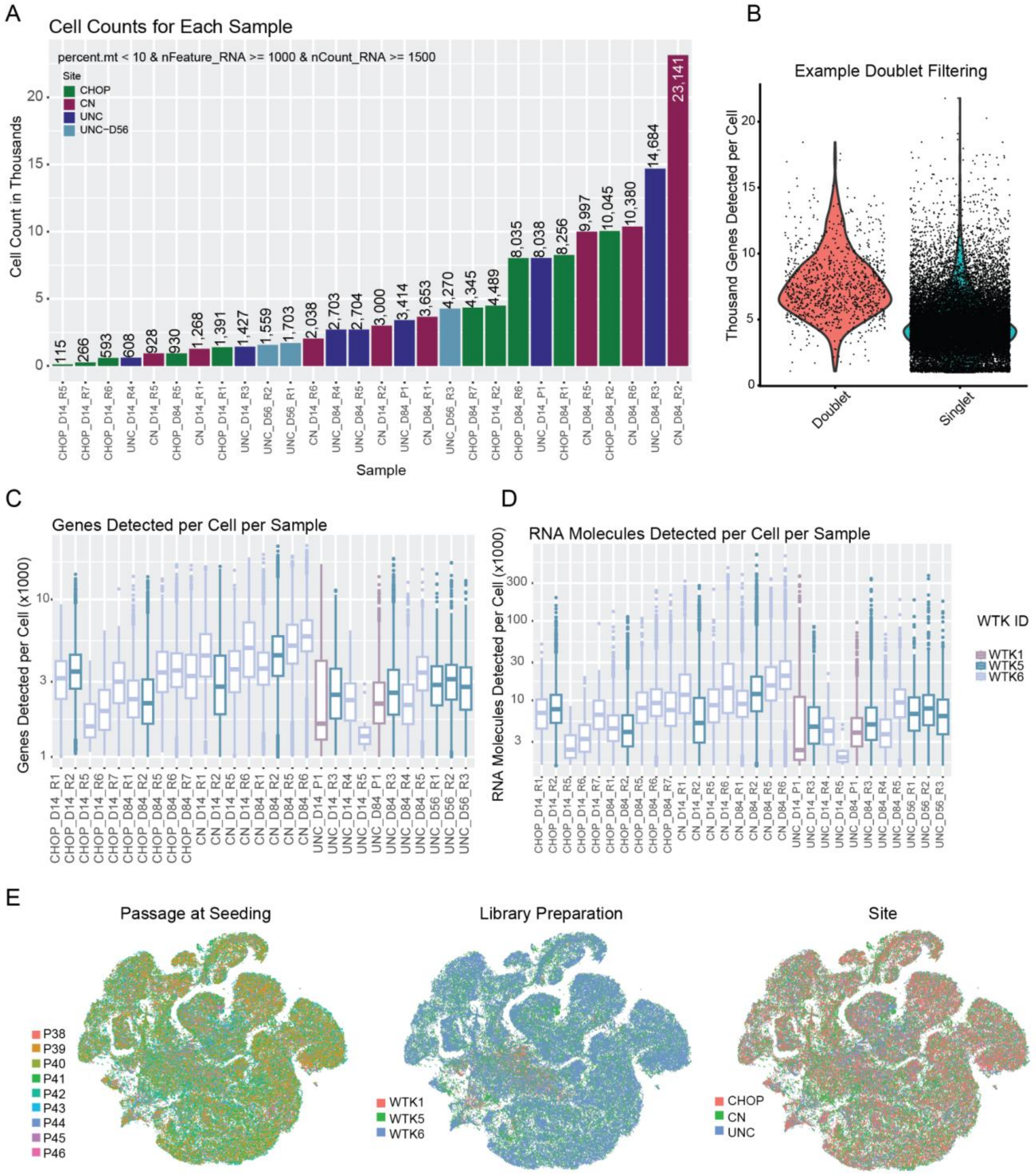

Supplementary Figure 1. Single Cell RNA Sequencing Quality Control. A) Final cell counts for each sample in scRNAseq after all quality control. One day 14 sample (UNC R5) was removed from the experiment because less than 100 cells remained following quality control. Fewer cells were loaded into the library preparation at day 14 due to smaller organoid size and likely reduced heterogeneity, so the fewer cells observed at this time point are expected. B) DoubletFinder finds a higher number of genes detected per cell in predicted doublets as

bioRxiv preprint doi: https://doi.org/10.1101/2023.07.28.550873; this version posted July 29, 2023. The copyright holder for this preprint (which was not certified by peer review) is the author/funder, who has granted bioRxiv a license to display the preprint in perpetuity. It is made available under aCC-BY 4.0 International license.

compared to predicted singlets. C) Average gene detected per cell and D) average RNA molecules detected per cell per sample, colored by Whole Transcriptome Kit (WTK) ID, which marks the library preparation batch. E) Colored tSNEs by technical batches and site.

bioRxiv preprint doi: https://doi.org/10.1101/2023.07.28.550873; this version posted July 29, 2023. The copyright holder for this preprint (which was not certified by peer review) is the author/funder, who has granted bioRxiv a license to display the preprint in perpetuity. It is made available under aCC-BY 4.0 International license.

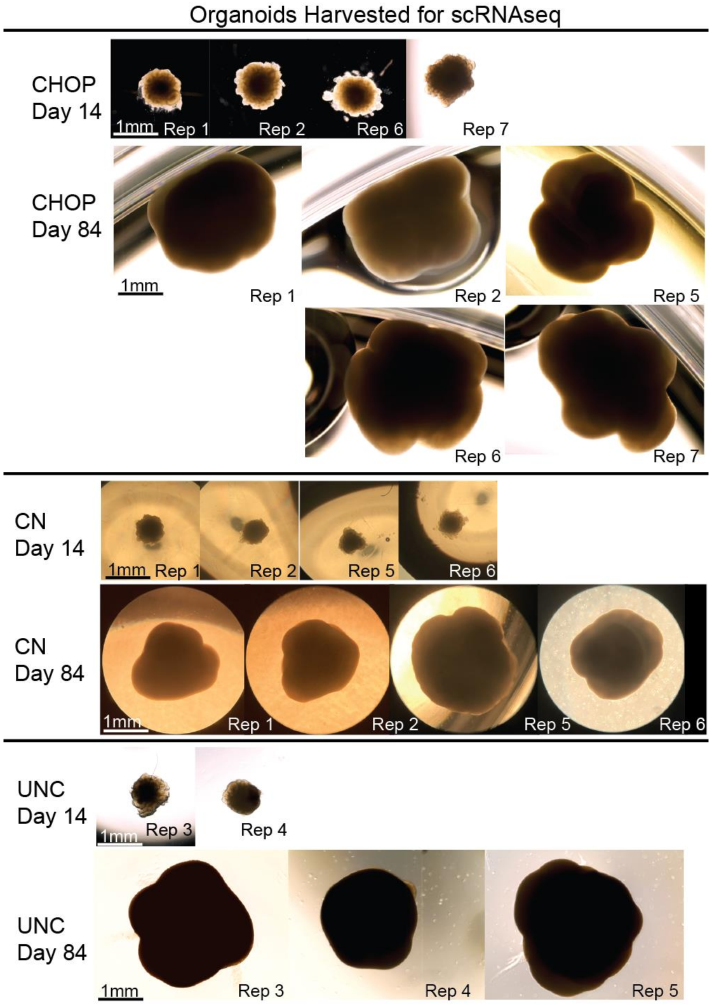

Supplemental Figure 2. Images of Organoids Used for scRNAseq. CN size measurements are estimated due to imaging warping, and the sizes of these organoids are not directly comparable with the other two sites. UNC Rep 5 is not shown because scRNAseq samples did not pass quality control. UNC pilot 1 did not have phase contrast images collected, so is also not shown.

bioRxiv preprint doi: https://doi.org/10.1101/2023.07.28.550873; this version posted July 29, 2023. The copyright holder for this preprint (which was not certified by peer review) is the author/funder, who has granted bioRxiv a license to display the preprint in perpetuity. It is made available under aCC-BY 4.0 International license.

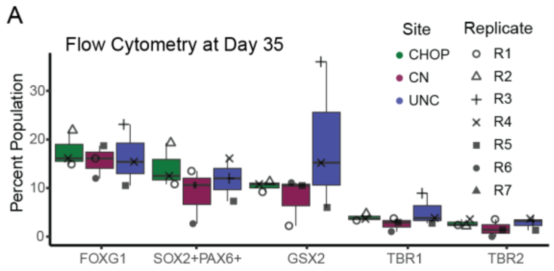

Supplemental Figure 3. Percent population expressing cortical and non-cortical cell type markers at day 35. FOXG1, SOX2+/PAX6+ and TBR2 indicate cortical progenitors. GSX2 is a marker for ventral forebrain progenitors (Corbin et al. 2000). TBR1 is a marker for early born cortical neurons. No significant differences across sites were detected.

bioRxiv preprint doi: https://doi.org/10.1101/2023.07.28.550873; this version posted July 29, 2023. The copyright holder for this preprint (which was not certified by peer review) is the author/funder, who has granted bioRxiv a license to display the preprint in perpetuity. It is made available under aCC-BY 4.0 International license.

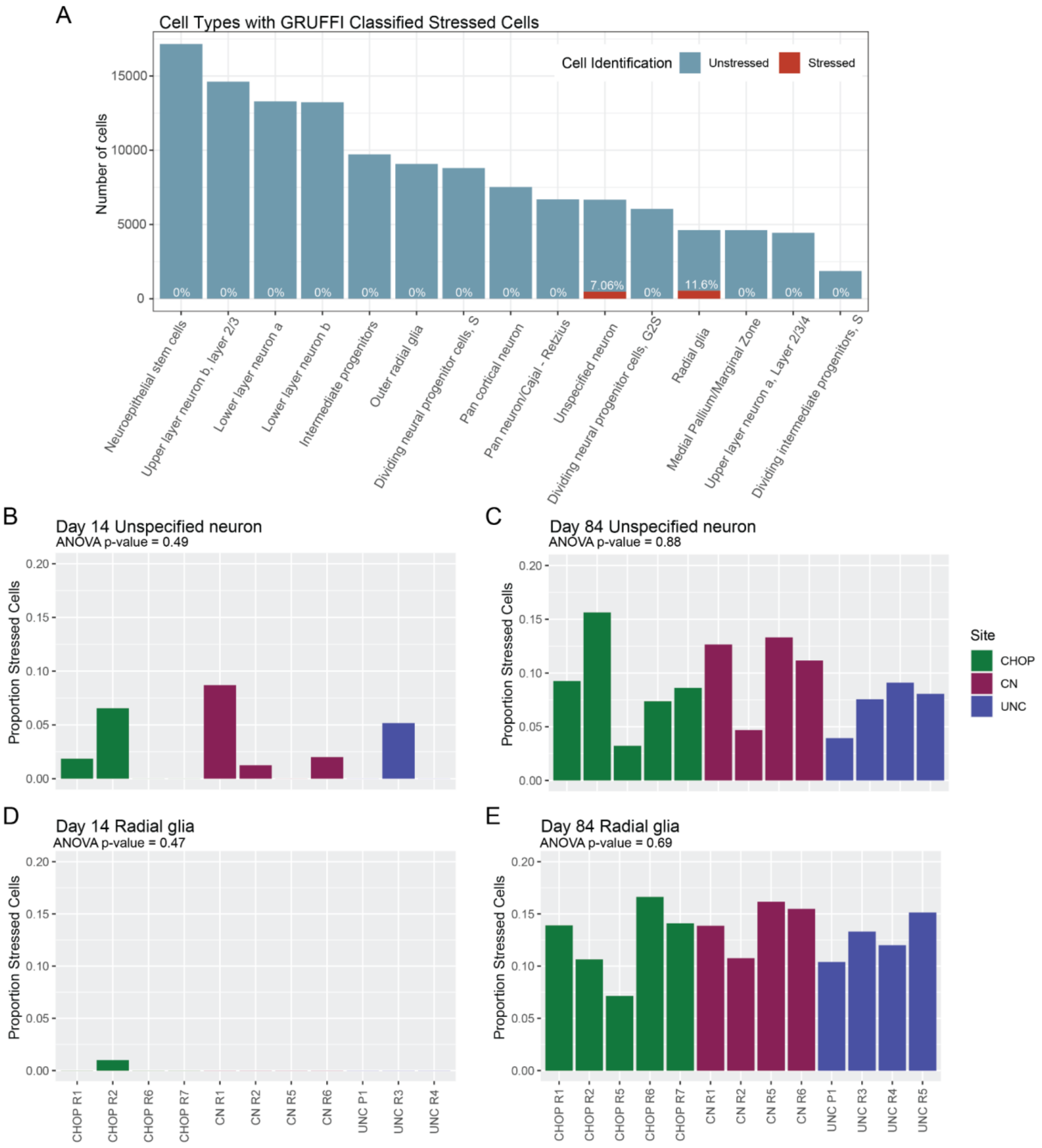

Supplementary Figure 4. Gruffi Identified Cells under Metabolic Stress. A) Percent stressed cells across cell types. B) Proportion of stressed cells in each scRNAseq experiment in B) day 14 unspecified neurons C) day 84 unspecified neurons, D) day 14 radial glia and E) day 84 radial glia. No significant differences were detected across sites.

bioRxiv preprint doi: https://doi.org/10.1101/2023.07.28.550873; this version posted July 29, 2023. The copyright holder for this preprint (which was not certified by peer review) is the author/funder, who has granted bioRxiv a license to display the preprint in perpetuity. It is made available under aCC-BY 4.0 International license.

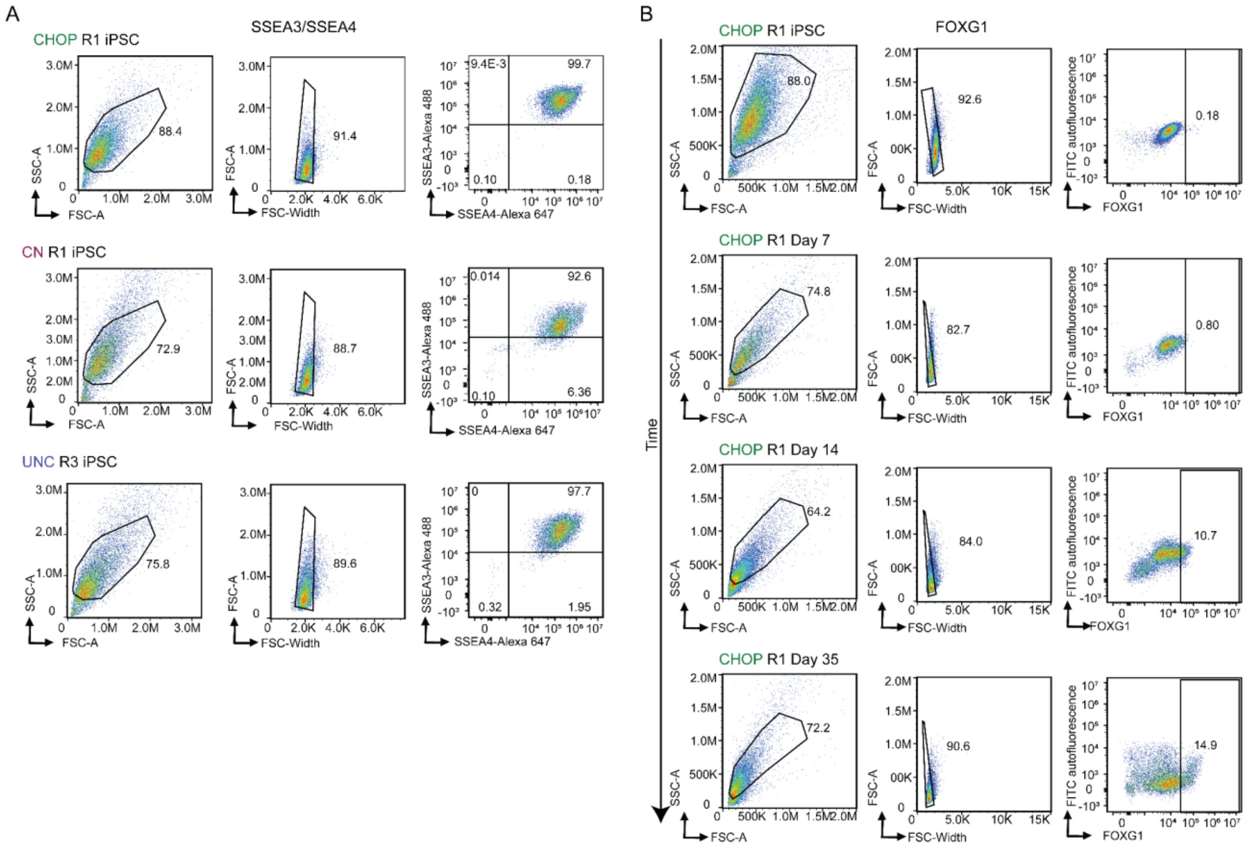

Supplemental figure 5. Example Flow Cytometry Gating. A) Gating for FOXG1 labeled cells in one sample across time. Gating was determined by day -1 immunoreactivity to FOXG1, which was negative. B) Gating for SSEA3/SSEA4 labeled cells from iPSC across all sites, which was determined relative to a no antibody control.

bioRxiv preprint doi: https://doi.org/10.1101/2023.07.28.550873; this version posted July 29, 2023. The copyright holder for this preprint (which was not certified by peer review) is the author/funder, who has granted bioRxiv a license to display the preprint in perpetuity. It is made available under aCC-BY 4.0 International license.

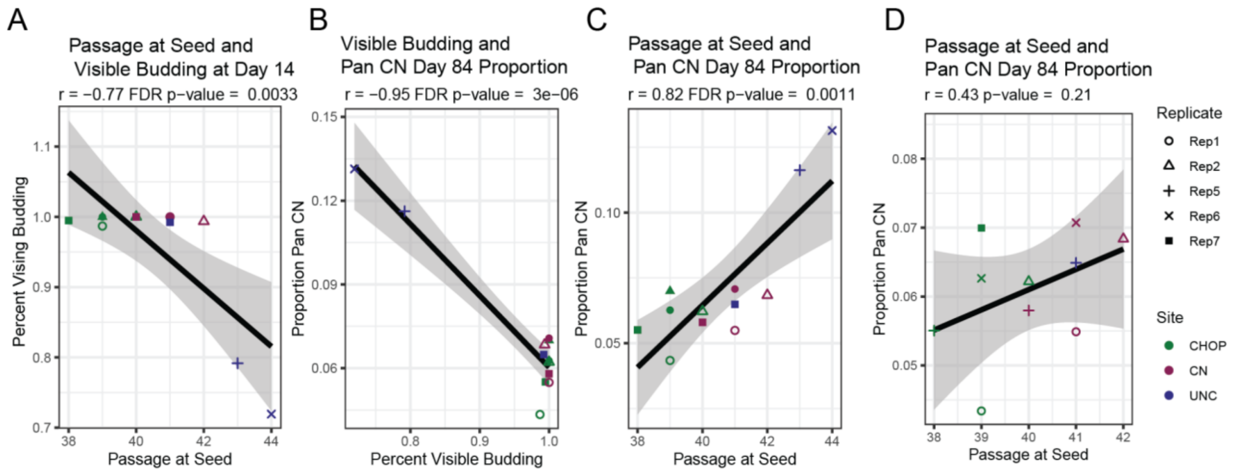

Supplemental Figure 6. Correlates to day 84 cell type proportions. A) Pearson's correlation between the two variables that correlated to the proportion of pan cortical neurons at day 84. Correlation between the proportion of pan cortical neurons at day 84 and B) percent visible budding or C) passage at seed. D) Correlation between passage at seed and the proportion of pan cortical neurons at day 84 when UNC R3 and UNC R4 outliers are removed.
